# Biomimetic three-layered membranes comprising (poly)-ε-caprolactone, collagen and mineralized collagen for guided bone regeneration

**DOI:** 10.1093/rb/rbab065

**Published:** 2021-11-24

**Authors:** Jingjing Wu, Mengyu Yao, Yonggang Zhang, Zefeng Lin, Wenwu Zou, Jiaping Li, Pamela Habibovic, Chang Du

**Affiliations:** 1 Department of Biomedical Engineering, School of Materials Science and Engineering, South China University of Technology, Guangzhou 510641, PR China; 2 National Engineering Research Center for Tissue Restoration and Reconstruction, South China University of Technology, Guangzhou 510006, PR China; 3 Key Laboratory of Biomedical Materials and Engineering of the Ministry of Education, and Innovation Center for Tissue Restoration and Reconstruction, South China University of Technology, Guangzhou 510006, PR China; 4 Department of Instructive Biomaterials Engineering, MERLN Institute for Technology-Inspired Regenerative Medicine, Maastricht University, Maastricht 6229 ER, the Netherlands

**Keywords:** bone regeneration, poly(caprolactone), mineralized collagen, electrospinning, artificial periosteum

## Abstract

The distinct structural properties and osteogenic capacity are important aspects to be taken into account when developing guided bone regeneration membranes. Herein, inspired by the structure and function of natural periosteum, we designed and fabricated using electrospinning a fibrous membrane comprising (poly)--ε-caprolactone (PCL), collagen-I (Col) and mineralized Col (MC). The three-layer membranes, having PCL as the outer layer, PCL/Col as the middle layer and PCL/Col/MC in different ratios (5/2.5/2.5 (PCM-1); 3.3/3.3/3.3 (PCM-2); 4/4/4 (PCM-3) (%, w/w/w)) as the inner layer, were produced. The physiochemical properties of the different layers were investigated and a good integration between the layers was observed. The three-layered membranes showed tensile properties in the range of those of natural periosteum. Moreover, the membranes exhibited excellent water absorption capability without changes of the thickness. *In vitro* experiments showed that the inner layer of the membranes supported attachment, proliferation, ingrowth and osteogenic differentiation of human bone marrow-derived stromal cells. In particular cells cultured on PCM-2 exhibited a significantly higher expression of osteogenesis-related proteins. The three-layered membranes successfully supported new bone formation inside a critical-size cranial defect in rats, with PCM-3 being the most efficient. The membranes developed here are promising candidates for guided bone regeneration applications.

## Introduction

Functional and cosmetic restoration of large bone defects caused by trauma, tumor removal or congenital malformations remains an important clinical challenge [1]. Transplantation of bone autograft and allograft is currently the gold-standard therapy to repair and regenerate damaged bone. However, the efficacy of this therapy is strongly impaired in the absence of periosteum, which is considered to contribute by about 70% to induction of *de novo* bone formation upon autograft implantation [2–4]. Furthermore, there is a direct correlation between periosteal stripping and bone nonunion [5]. The regenerative capacity of periosteum has been employed to heal critical-sized bone defects [6–8]. Therefore, preservation of periosteum or implantation of an artificial periosteum is suggested to improve the osteogenic capacity and bone regenerative potential of bone grafts (substitutes) [7].

Periosteum is a thin, dense, osteogenic tissue enveloping the outer surface of the cortical bone and is comprised of an inner osteogenic cambium layer and an outer fibrous layer [9]. The osteogenic cambium layer houses osteoblasts, osteogenic precursors, periosteal stem cells and thin collagen fibers [10]. Comprehensive histological studies of the outer layer have shown that it comprises two parts. The superficial portion is generally inelastic and relatively cell poor but is the most highly vascularized part of the periosteum. The deep portion of the outer layer is elastic, owing to the presence of elastin and collagen fibers [11]. Mimicking the specific structural and functional features of natural periosteum matrix is considered a promising strategy to obtain an artificial periosteum that may aid the bone repair and regeneration strategies. Important requirements for the biomaterial used to produce artificial periosteum or guided bone regeneration membrane are degradability *in vivo* and the ability to support new bone formation at the desired site. Several biomaterials and fabrication methods have been exploited to produce guided bone regeneration membrane that recapitulates one or other features of natural periosteum [12–14]. These studies focused on the delivery of progenitor cells, inorganic component and/or growth factors to repair the bone defect using hydrogel or single-layer membrane. It is still a challenge to emulate multilayered structure and multifunction of the natural periosteum.

Electrospinning is a promising method to fabricate fibrous membranes with natural extracellular matrix (ECM)-like structural features. Various materials, predominantly polymers, can be processed using electrospinning and bioactive components can be added to inert matrices [15, 16]. (Poly)-ε-caprolactone (PCL), an electrospinnable semicrystalline polymer approved for use in humans by the Food and Drug Administration, has been widely used in biomedical applications, including regenerative medicine, for its safety, availability and good mechanical properties. However, the low surface energy (high hydrophobicity) of PCL has been shown to inhibit cell attachment and proliferation [17], demonstrating the need for surface treatments, e.g. by plasma [18], or addition of bioactive compounds, including calcium phosphate, bioglass or growth factors. In the case of blends and composites, the interaction between the filler material and the PCL matrix has been shown to affect the physicochemical properties and mechanical stability [19–21]. Tsai *et al*. [22] demonstrated that strontium-substituted hydroxyapatite (HA)-containing PCL membrane can significantly promote osteogenic differentiation and mineralization of osteoblast-like cells. In another study, Ebrahimi *et al.* [23] fabricated a PCL/poly(vinyl alcohol) membranes containing human endometrial stem cells (hEnSCs) and metformin to facilitate new bone formation. Gao *et al.* [24] developed scaffolds comprising collagen, bioactive glass and PCL to accelerate wound healing by enhancing angiogenesis.

Type I collagen (Col) is the main organic constituent of bone matrix and periosteum and is responsible for the toughness of the tissue and for providing a favorable microenvironment to cells [25]. Col is therefore an extensively used biomaterial in bone regeneration [26].

Mineralized collagen (MC), a fibrous collagen matrix containing calcium phosphate (CaP), is another interesting material in the context of bone regeneration, as it emulates the composition and hierarchical structure of natural bone tissue. Previous studies by our and other groups showed beneficial effects of MC in mesenchymal stromal cell differentiation and bone regeneration in preclinical and clinical studies [27–30]. Combining Col and MC with PCL is therefore an attractive approach to develop bioactive guided bone regeneration membranes.

In addition to the selection of biomaterials, architectural design is also critical when developing artificial periosteum. In this study, inspired by the layered structure of periosteum and specific function of each layer [31], a membrane was created that comprises three layers, i.e. a layer of PCL, a layer of PCL/Col and a layer of PCL/Col/MC, made by electrospinning. Hydrophobic PCL layer was selected as the outer layer to act as a barrier preventing the surrounding connective tissues from growing into the bone defect area. PCL/Col acted as the middle layer, endowing the construct with elastic properties. The PCL/Col/MC inner layer was created to offer a favorable microenvironment for recruited cells to differentiate into the osteogenic lineage and support new bone formation. The different proportions of PCL, Col and MC in the inner layer were comparatively studied. The membranes underwent thorough physicochemical characterization and the biological response to the membranes was studied *in vitro* and *in vivo*.

## Materials and methods

### Raw materials

Type-I collagen (Col) from bovine tendon was obtained from the Beijing Allgens Medical Science and Technology Co. Ltd. All other chemicals were purchased from Sigma-Aldrich and were used without further purification. PCL (Mn = 80 000) was placed in a vacuum oven at 40°C for at least 24 h to remove residual water.

### Fabrication of PCM composite membrane

The MC powder was prepared as previously reported [32]. Briefly, the calcium chloride (CaCl_2_) and sodium phosphate monobasic (NaH_2_PO_4_) solution (Ca/*P* = 1.67) were dropwise added into acidic Col solution under stirring. The pH value of the prepared solution was adjusted to 7.4 with sodium hydroxide (NaOH) and stirred for 48 h. The formed MC was then harvested by centrifugation. After washing with deionized water for three times, MC was lyophilized and ground into powder for further use. The grain size of MC powder was between 10 and 100 μm.

The gradient membrane was fabricated using electrospinning technology. Solution of PCL (10% w/w), 3:1 PCL/Col (7.5% PCL, 2.5% Col, w/w), 2:1:1 PCL/Col/MC (5% PCL, 2.5% Col, 2.5% MC, w/w), 1:1:1 PCL/Col/MC (3.3% w/w of each component) and 1:1:1 PCL/Col/MC (4% w/w of each component) were obtained by dissolving PCL, Col and MC in the desired ratio in hexafluoroisopropanol (HFIP) under stirring for 12 h. For the preparation of three-layered membranes, PCL, PCL/Col and PCL/Col/MC solutions were loaded in 10 ml syringes and electrospun successively under the control of syringe pumps. PCL (10% w/w) and 3:1 PCL/Col (7.5% PCL, 2.5% Col, w/w) were used in each group as outer layer and middle layer, respectively. 2:1:1 PCL/Col/MC (5% PCL, 2.5% Col, 2.5% MC, w/w), 1:1:1 PCL/Col/MC (3.3% w/w of each component) and 1:1:1 PCL/Col/MC (4% w/w of each component) were used as the inner layer and denoted as PCM-1, PCM-2 and PCM-3, respectively (Table 1). To ensure integration between the layers, electrospinning of the next solution was started before that of the previous solution was stopped. The voltage of 15 kV was applied and the flowing rate was maintained at 10 μL min^−1^. The multilayer fibrous membrane for *in vivo* animal study was collected on aluminum foil placed on collector and single-layer sheets for cell culture were collected on round glass coverslips (12 mm in diameter) after sterilization with 75% ethanol and UV overnight.

### Membrane characterization

Morphology of the membranes was observed by field-emission scanning electron microscopy (FE-SEM, NovaNanoSEM430, FEI) equipped with an energy-dispersive X-ray spectrometer (EDS, X-Max Extreme, Oxford). Surface chemical composition of the samples was investigated using a Fourier transform infrared spectrophotometer (ATR-FTIR, Alpha-E, Bruker). X-ray diffraction (XRD, MiniFlex 600, Rigaku) analysis was performed to determine the crystallinity of the samples. Wettability of the samples was evaluated by the water contact angle measurement equipment (OCA15, Dataphysics, Germany), and six parallel tests were carried out on the different areas of each sample.

The mechanical properties of the composite membranes were studied using a dynamic mechanical analyzer (Q800, TA Instruments, USA). Specimens were cut into small strips of 20 mm × 5 mm with the thickness of 0.5 mm and fixed on the testing machine. Then samples were stretched at the speed of 1 N/min at room temperature and stress–strain curves, tensile stress, Young’s modulus and elongation at break were determined. A minimum of five samples was tested.

To test the swelling ratio of the membranes, samples were immersed in phosphate-buffered saline (PBS) for 12 h and the mass weight before and after soaking was recorded in 15 min time intervals. The swelling ratio was calculated using the following equation:

$$\text{Swelling}\;\left(\%\right)=\frac{Ww-Wd}{Wd}\times 100$$

where W_d_ is the weight of dry sample and W_w_ is the weight of samples after swelling.

### *In vitro* biological performances

#### Cell culture, morphology and cell viability

Human bone marrow-derived stromal cells (hBMSCs) and hBMSCs with stable green fluorescent protein (GFP-hBMSCs) were purchased from Cyagen Biosciences and cultured in basic Dulbecco’s modified Eagle medium supplemented with 10% fetal bovine serum, 2 mM glutamine, 100 U/ml penicillin and 100 mg/ml streptomycin (Cyagen Biosciences, Guangzhou, China) in a humidified atmosphere of 95% air and 5% carbon dioxide (CO_2_) at 37°C. The cells were cultured on PCL and PCM membranes with a diameter of 12 mm inside 24-well plate (Costa), using wells without a material as a control.

Cell attachment and morphology (*n* = 5) were analyzed by SEM and laser scanning confocal microscopy (TCS SP8, Leica, Germany). Prior to the SEM analysis, hBMSCs were seeded on the materials at a density of 1 × 10^4^ ml^−1^ and cultured for 2 days, dehydrated by a gradient ethanol (C_2_H_5_OH) series and dried. GFP-hBMSCs were seeded at a density of 2 × 10^4^ ml^−1^ and cultured for 2 days, washed with PBS, fixed with 4% paraformaldehyde and observed directly using laser scanning confocal microscope.

Quantitative cell proliferation (*n* = 3) analysis was performed by Cell Counting Kit-8 (CCK-8, Dojindo, Japan) according to the manufacturer’s instructions. Briefly hBMSCs were seeded on the sterilized materials or the empty wells at a density of 2 × 10^4^ ml^−1^. At days 1, 4 and 7, the samples were washed with PBS for three times before adding a mixture of 200 µl DMEM medium and 20 µl CCK-8 reagent to each well. After incubating for 1 h, the medium was transferred to 96-well plate and the absorbance was measured at the wavelength of 450 nm with a microplate reader (Thermo scientific 3001, USA).

To test integration between the layers and cell distribution through the three-layered membranes, the materials were immersed into hBMSCs suspension with a density of 10^6^ ml^−1^ inside 24-well plate. After incubation for 14 days, the samples were fixed with 4% paraformaldehyde, dehydrated by a gradient ethanol series and embedded in paraffin wax. Subsequently, cross section was made at 5 μm for histologic analysis. The sections were deparaffinized and rehydrated before staining. For histologic analysis, sections were stained with hematoxylin-eosin (H&E) and observed using digital slide scanner (P250 FLASH, 3DHISTECH, Hungary).

#### Cell migration

The migration of cells on the materials (*n* = 3) was detected using the *in vitro* CytoSelect™ 24-Well Wound Healing Assay Kit from Cell Biolabs Inc. (San Diego, CA). Briefly, GFP-hBMSCs were seeded at a density of 2 × 10^5^ ml^−1^ on the different biomaterials through the inserts. After 1 day, the inserts were carefully removed from the wells to generate a 0.9 mm wound gap. The cells on the materials were then allowed to migrate into the gap for 24 and 48 h. After fixation, wound gaps were visualized with a light microscope (Nikon Instruments Europe BV, the Netherlands). The number of cells migrated to the cell-free gap was counted by three blinded observers.

#### Osteogenic differentiation of hBMSCs

Enzymatic alkaline phosphatase (ALP) activity (*n* = 5) was quantitatively assessed using an ALP assay kit (P0012S, Beyotime Company, China) according to manufacturer’s instructions. hBMSCs were seeded on the materials at a density of 5 × 10^4^ ml^−1^ and cultured for 3, 7, 14 and 21 days, then washed three times with PBS and incubated in RIPA lysis buffer (P0013, Beyotime Company, China) for 2 h on ice; 50 µl of the cell lysate of each sample was mixed with 50 µl ALP buffer for 30 min at 37°C. The reaction was stopped by adding 100 µl of reaction stop solution. The relative ALP activity was measured at absorbance wavelength of 405 nm using a microplate reader (Thermo scientific 3001, USA). Calibration curve was made using different concentrations of p-nitrophenyl. The ALP activity was normalized by the corresponding total protein content, which was determined using a BCA Protein Assay Kit (Thermo scientific NCI3225CH, USA) according to the manufacturer’s instructions.

The expression of osteogenic proteins osteocalcin (OCN), osteopontin (OPN), type I collagen (COL-I) and ALP (*n* = 3) was detected by Western blot. In brief, the cells were seeded on the materials at a density of 5 × 10^4^ ml^−1^ and cultured for 14 and 21 days. The cells were lysed and the total protein content was determined as described above. Then, proteins were separated by SDS-PAGE and transferred to a polyvinylidene fluoride (PVDF) membrane (Millipore, MA, USA), which was first incubated with 1% BSA blocking solution for 60 minutes and then with antibodies targeting ALP (1:500, Abcam, USA), OPN (1:500, Abcam, USA), OCN (1:500, Abcam, USA), COL-I (1:500, Abcam, USA) and GAPDH (1:2500, Abcam) at 4°C overnight. The membrane was washed and incubated with horseradish peroxidase-conjugated secondary antibody (Abcam, USA) at room temperature for 1 h, and the protein expression was visualized using a Bio-Rad Chemidoc XRS system.

### *In vivo* study in rats

#### Surgical procedure

The animal experiments were approved by the Animal Experimental Center of Guangdong Pharmaceutical University. Adult female Sprague Dawley rats aged 8 weeks (220 ± 20 g, *n* = 6) were purchased from Guangdong Medical Laboratory Animal Center (Guangzhou, China). The three-layered membranes were cut into wafers with a diameter of 5 mm and sterilized with ^60^Co-gamma ray at a dose of 15 kGy.

The animals were anesthetized by intraperitoneal injection of pentobarbital (Nembutal 2 mg/100g). The procedures were performed in sterile conditions. The skin of the skull was shaved and a 2.5 cm long incision was made. The incised skin was spread by a dilator to fully expose parietal bone and the soft tissue was removed from the surface with blunt knife. A hole with a diameter of 5 mm diameter and a thickness of 0.5 mm was created in parietal bone with a surgical trephine burr (Bernal Dental, Britain). The defect was washed with sterile saline solution to cool the defect area and remove bone debris. Two defects were created in the skull of each animal and the defects were filled with PCL, PCM1, PCM2, PCM3 membranes or left empty. For each group, six animals were sacrificed after 4 weeks and another six after 12 weeks, and calvarial bones were collected and stored in 4% paraformaldehyde solution immediately.

#### Micro-computed tomography evaluation

Micro-CT (SKYSCan1176, Germany) was used to investigate bone formation. The analysis was carried out at a current of 313 μA, a voltage of 80 kV and the image pixel size was set at 17.4 μm. The collected data were used to reconstruct 3D tomographic images and bone volume-to-tissue volume ratio (BV/TV) and bone mineral density (BMD) were analyzed based on micro-CT images.

#### Histological observation

After fixation in 4% paraformaldehyde for 48 h, the samples were immersed in neutral 10% ethylene diamine tetraacetic acid (EDTA) solution for a period of 4 weeks with refreshment every 2 d for decalcification. Subsequently, samples were dehydrated through a graded alcohol series (70–100%) and then embedded in paraffin. The samples were cut into 5 µm sections, deparaffinized and rehydrated and further stained with hematoxylin–eosin solution (H&E) and Masson’s trichrome staining to visualize tissue formation.

### Statistical analysis

All analyses were performed on at least three independent samples. Data are expressed as mean ± standard deviation (SD). Statistical analysis was performed using SPSS16.0 software, and one-way ANOVA with a Tukey’s post hoc test was used to determine the significant differences between groups. Differences with *P* < 0.05 were considered to be statistically significant.

## Results and discussion

**Figure 1.**
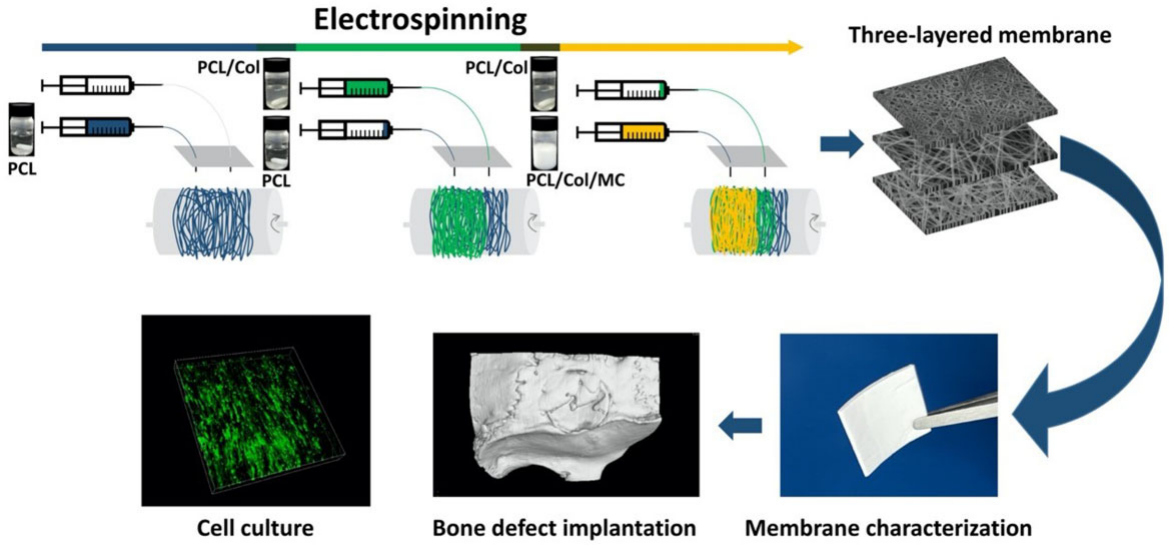
Schematic of fabrication and evaluation of three-layered fibrous membranes.

As schematically shown in Fig.  1, this study aims to develop a novel three-layered guided bone regeneration membrane, inspired by the structure and function of the natural periosteum and to investigate the response of hBMSCs to the membranes as well as their bone regenerative potential *in vivo*.

### Characterization of membranes

**Figure 2.**
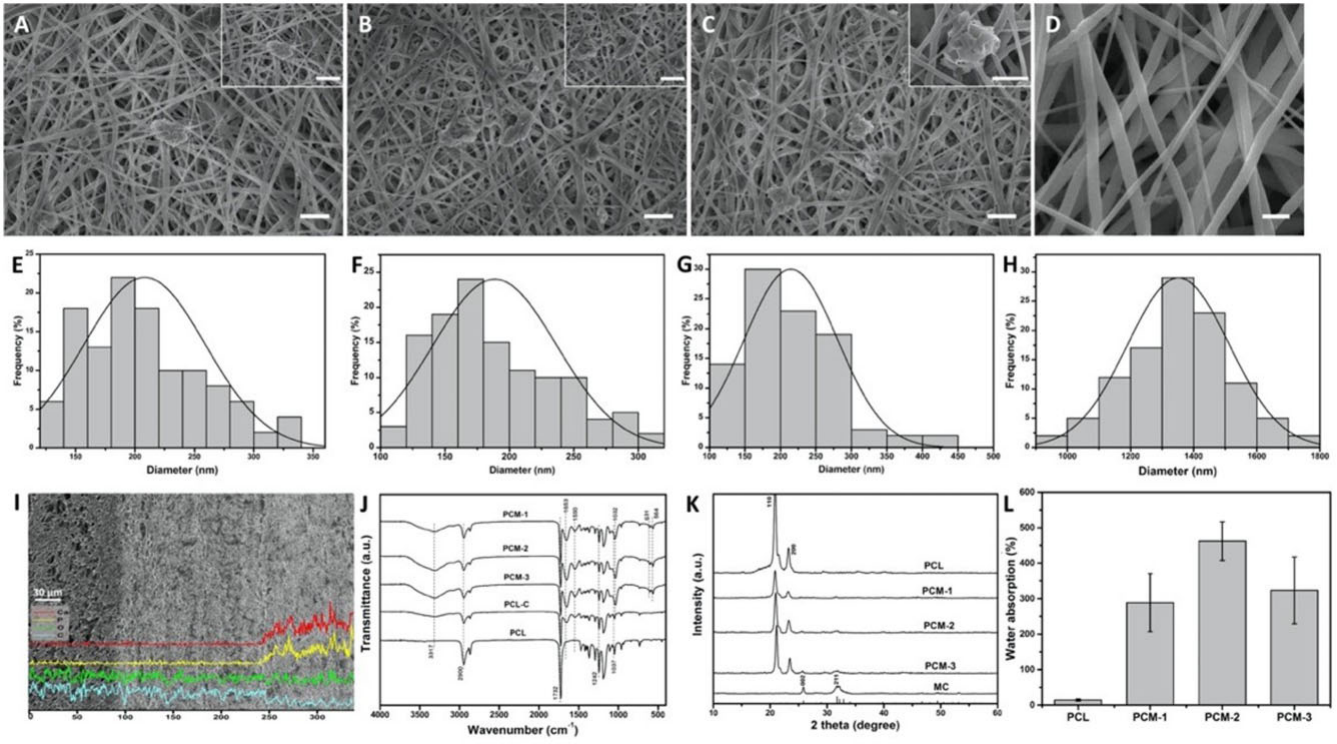
SEM Images and corresponding fiber diameter histograms of the inner layer of PCM-1 (**A**, **E**), PCM-2 (**B**, **F**), PCM-3 (**C**, **G**); and the outer PCL layer (**D**, **H**), scale bar: 2 µm; SEM image and EDS element analysis of the cross-section of a PCM membrane (**I**); FTIR spectra (**J**), XRD pattern (**K**) and water absorption ability (**L**) of the different membranes.

Figure  2 summarizes the physicochemical and structural characterization of the membranes. SEM images (Fig.  2A–D) illustrate the surface structure of the inner layer of PCM-1, PCM-2, PCM-3 and the outer PCL layer, respectively. Both the inner and the outer layer exhibited a fibrous structure resulting from electrospinning. The PCM layer, comprising PCL, Col and MC in different ratios showed a dense fibrous network with thin fibers having a rough surface, in contrast to the PCL network, which appeared more loose and comprising thicker fibers with a smooth surface. Throughout the PCM network, particles of MC were observed. The quantification of the fiber diameter (Fig.  2E–H) confirmed the difference in fiber thickness; the fiber diameter was 208.13 ± 51.1 nm, 188.94 ± 48.42 nm and 214.76 ± 65.72 nm for PCM-1, PCM-2 and PCM-3, respectively, while that of the outer PCL layer was 1351.91 ± 161.18 nm (Image-Pro Plus, v.6.0, *n* = 100).

Figure  2I shows an SEM image of the typical cross-sectional morphology of a PCM membrane, with consecutive outer PCL layer, middle PCL/Col layer and inner PCL/Col/MC layer. The middle and the inner layer appear closely bonded without a clear boundary between them. EDS spectrum showed the distribution of elements and the presence of calcium (Ca) and phosphorus (P) in the inner layer of the PCM membrane. The middle layer and the inner layer were different in composition, but similar in morphology.

The FTIR spectra of PCL, PCM-1, PCM-2 and PCM-3 are shown in Fig.  2J. The characteristic bands of PCL were detected at 1732 cm^−1^ (C–O–C stretching), 1242 cm^−1^ (C–O stretching) and 1037 cm^−1^ (C–O stretching). MC and Col have common characteristic peaks probed at 3317 cm^−1^ (amide A), 1653 cm^−1^ (amide I), 1550 cm^−1^ (amide II) and 1032 cm^−1^ (C-OH). Another two bands of MC at 631 cm^−1^ and 564 cm^−1^ were attributed to the O–H absorption band and ${\text{PO}}_{4}^{3}$^−^ stretching band. The XRD patterns of the materials (Fig.  2K) showed the diffraction peaks characteristic of PCL at approximately 2 theta values of 21.5° and 23.9° in all sample. PCM-1, PCM-2 and PCM-3 showed characteristic HA peaks, indicating the incorporation of MC. Furthermore, the relative peak intensity of PCM-1, PCM-2 and PCM-3 increased with the increasing MC content. These results signified that a series of PCL/Col/MC ECM-like three-layered composite membranes were prepared successfully.

The rate of water absorption is considered important in the context of biomaterials for tissue engineering and regenerative medicine because the uptake of physiological fluids and delivery of nutrients and metabolites to cells occur through the substrate. From Fig.  2L, it can be seen that membranes consisting of PCL only exhibited a relatively low water absorption capacity of 14%. Water absorption by the three-layered PCM membranes was significantly higher with 288%, 462% and 323% for PCM-1, PCM-2 and PCM-3, respectively. The increase of water absorption capacity was not accompanied by the change in the thickness of the membranes.

**Figure 3.**
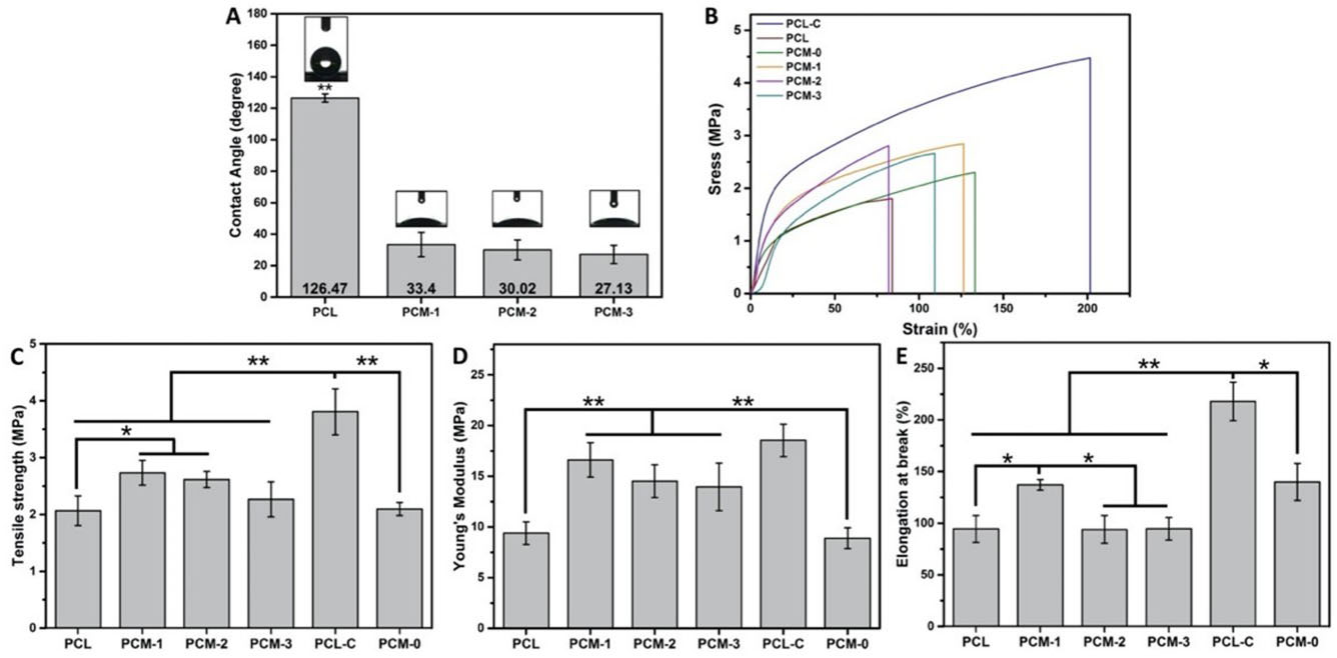
Water contact angle measurement of the inner, PCM layer and the outer PCL layer of the three-layered PCM membranes (**A**); representative stress–strain patterns of tensile properties of membranes (**B**); tensile strength (**C**); Young’s module (**D**); elongation at break (**E**). (*P < 0.05, **P < 0.01).

Figure  3A shows the contact angle of the outer PCL layer and the inner PCM layer with different ratio of its constituents. The contact angle of the PCL layer was 126.47 ± 2.57, indicating its hydrophobic nature due to the CH_3_ group. In contrast, the contact angle of the inner layer comprising PCM-1, PCM-2 and PCM-3 was 33.4 ± 13.79, 30.02 ± 12.31, 27.13 ± 5.83, respectively showing a significant increase of hydrophilicity by the addition of Col and MC to PCL.

The mechanical properties of the membranes are shown in Fig.  3B–E, including stress–strain curve, tensile stress, Young’s modulus and elongation at break. Single-layer samples of PCL, PCL-Col (PCL/Col, (3/1), (%, w/w)) and PCM-0 (PCL/Col/MC, 4/4/4, (%, w/w/w)) were used for comparison. Regarding the comparison of single-layer samples, the tensile stress, Young’s modulus and elongation at break of PCL-Col were about two times higher than that of PCL and a single-layer membrane with a composition of PCM-0.Enhancement of ultimate tensile strength of PCL by addition of Col is in close agreement with literature [33]. There was no obvious difference in tensile strength and Young’s modulus before and after incorporation of MC. The performance in tensile strength and Young’s modulus of the single-layer PCL-Col was the highest among all samples and significantly higher than that of the three-layered membranes. The mechanical properties of the three-layered membranes were higher as compared to single-layered membranes consisting of PCL and PCM-0. Comparing the three three-layered membranes, the tensile strength and Young’s modulus decreased in the order PCM-1, PCM-2 and PCM-3, showing than the increase in the amount of MC (and Col), together with a decrease in the amount of PCL had a negative effect on the tensile properties of the membranes. Earlier work has shown that natural periosteum exhibits anisotropic mechanical properties, depending on the direction of testing. The Young’s modulus of PCM membranes lies between the values of the elasticity modulus of axially oriented (1.93 ± 0.14 MPa ∼ 25.67 ± 6.87 MPa) and circumferentially oriented (4.41 ± 1.21 MPa) natural periosteum [34]. It should be noted that the guided bone regeneration grafts are rarely exposed to high tensile stress after implantation at the defect sites. Therefore, PCM membranes exhibited satisfactory mechanical properties.

### *In vitro* biological performance

#### Attachment, proliferation and migration of hBMSCs

**Figure 4.**
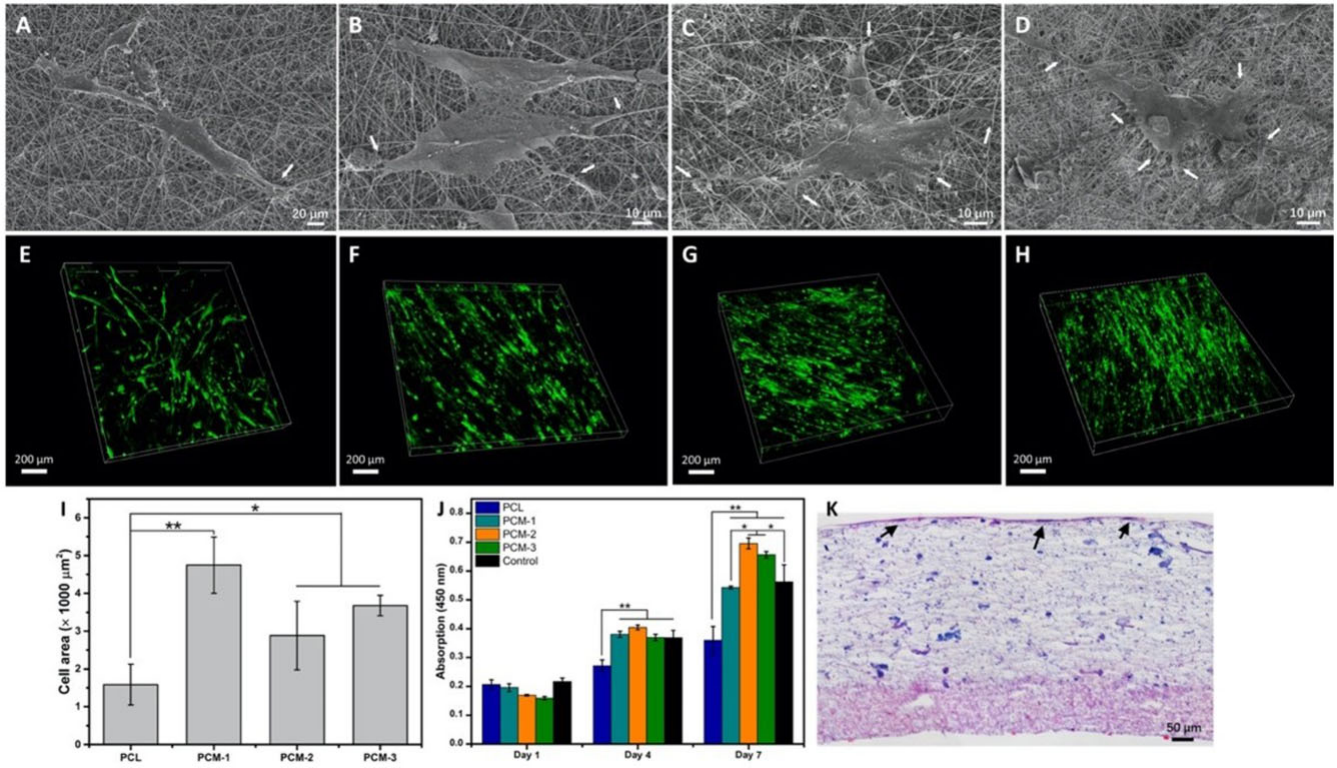
Morphology of hBMSCs on the inner layer of the different fibrous membranes after 2 days of culture. SEM images cells on PCL (**A**), PCM-1 (**B**), PCM-2 (**C**), PCM-3 (**D**), white arrows indicate the philopodia; the fluorescence images of GFP-hBMSCs on PCL (**E**), PCM-1 (**F**), PCM-2 (**G**), PCM-3 (**H**). Cell area of hBMSCs on different membranes after 2-day culture (A) (*n* = 10); cell proliferation of hBMSCs cultured on different membranes for up to 7 days (J); the H&E staining of cross-section images of hBMSCs cultured on PCM trilayer membrane for 14 days (K), black arrows indicate the cell layer (*P < 0.05, **P < 0.01).

The attachment and morphology of hBMSCs on the PCM membranes were analyzed by SEM and fluorescence microscopy. As is shown in Fig.  4, after 48 h of culture, more cells were observed on PCM-1, PCM-2 and PCM-3 as compared to the PCL membrane. SEM images (Fig.  4A–D) exhibited a comparatively more spread morphology of cells and presence of more filopodia on PCM membranes. Moreover, the qualitative observation of fluorescence microscopy images (Fig.  4E–H) suggested that the cells cultured on PCM membranes, and in particular on PCM-2 and PCM-3 were more unidirectionally oriented, while the orientation of hBMSCs on PCL was more random. The quantification of cell area (Fig.  4I) confirmed significantly more pronounced spreading of cells on PCM, and in particular PCM-1 membrane, plausibly due to a sparse distribution of MC particles that supported cell attachment.

Cell proliferation assay showed an increase in cell number between day 1 and day 7 on all materials (Fig.  4J). At days 4 and 7, significantly higher cell numbers were observed on PCM membranes as compared to PCL, with PCM-2 showing the highest values. Together, these results indicated that PCM membranes supported hBMSCs attachment and proliferation.

Figure  4K showed the histological sections of three-layered PCM membranes after 14-day hBMSCs culture. A thick layer of cells was observed inside the inner membrane layer. The three layers remained continuous and intact, and the boundary between the middle and the inner layer was still not obvious, which is consistent with the SEM results.

**Figure 5.**
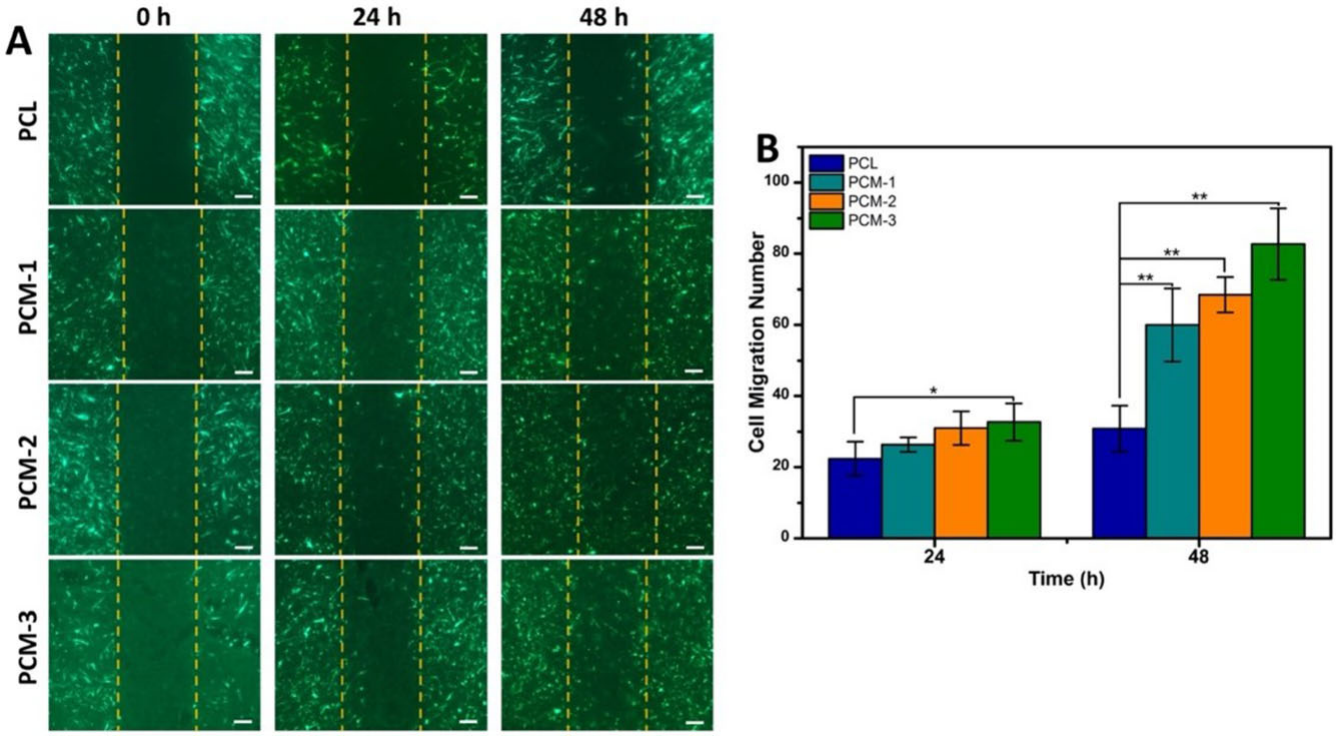
(**A**) *In vitro* migration of GFP-hBMSCs on PCM membranes in the wound healing assay at 0, 24 and 48 h, scale bars = 200 µm; (**B**) quantification of cell migration.

To investigate the ability of different PCM membranes to support GFP-hBMSCs migration, an *in vitro* wound-healing assay was used to create a cell-free gap. As is shown in Fig.  5A, all the samples supported cell migration and cell number in the gap increased between 24 and 48 h. The number of migrated cells in different groups after 24 h, as counted by blinded observers, was 23 ± 5, 27 ± 2, 31 ± 5 and 33 ± 6 cells for PCL, PCM-1, PCM-2, PCM-3, respectively. Differences were statistically significant between PCL and PCM-3. After 48 h, the number of migrated cells was 31 ± 6, 60 ± 11, 68 ± 4 and 83 ± 10 for PCL, PCM-1, PCM-2, PCM-3, respectively. All PCM membranes showed significantly higher values than PCL. From this, it can be inferred that more cells migrated on the membrane with higher Col-MC content.

### Osteogenic differentiation of hBMSCs

**Figure 6.**
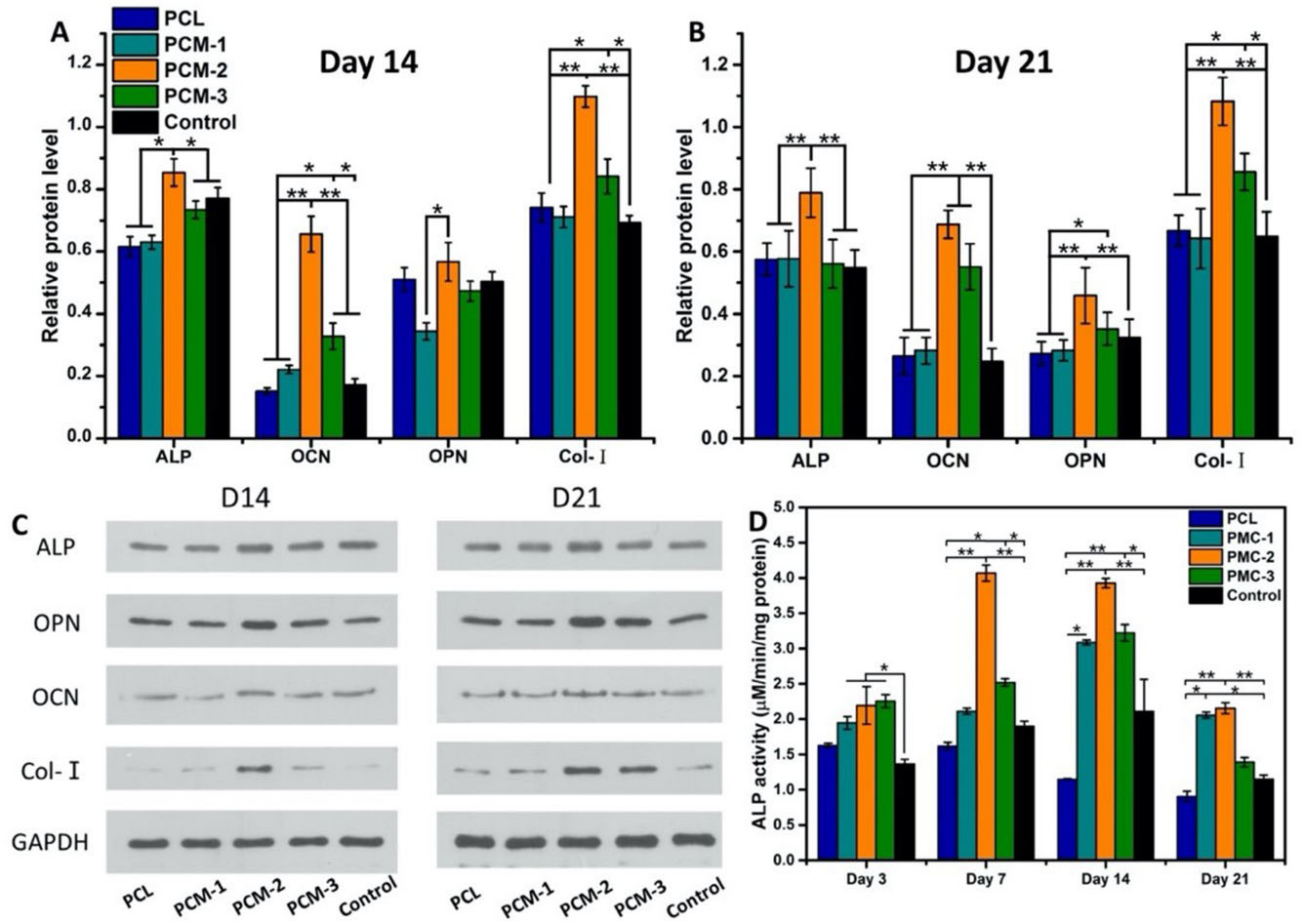
Protein expression (ALP, OPN, OCN and Col-I by hBMSCs cultured on the different membranes for 14 and 21 days (**A–C**)). Normalized ALP activity of hBMSCs cultured on samples for up to 21 days (**D**) (*P < 0.05, **P < 0.01).

Efficient differentiation of MSCs toward the osteogenic lineage is considered imperative for successful and rapid healing of large bone defect [35]. ALP is frequently used as an early marker of osteogenic differentiation. The overall trend was that the ALP activity increased between day 3 and 7, remained relatively high up to day 14 and then decreased again. Quantitative evaluation of the ALP activity of hBMCS cultured on the different membranes in basic medium, i.e. in the absence of soluble stimulators of differentiation, showed that PCM membranes supported the osteogenic differentiation of the cells (Fig.  6D). At all time points, cells on PCM membranes showed significantly higher ALP activity values than those cultured on the tissue culture plastic control. Similarly, at days 7, 14 and 21, the ALP activity on the PCM membranes was significantly higher than on PCL. In addition, at days 7 and 14, PCM-2 membrane exhibited significantly higher ALP activity than the PCM-1 and PCM-3 membrane. After 21 days, the ALP activity was similar for PCM-1 and PCM-2, and significantly higher than for PCM-3.

Further investigation of osteogenic proteins was performed by Western blot after 14 and 21 days of culture (Fig.  6). The positive effect of PCM-2 on the ALP activity of hBMSCs was confirmed by the Western blot analysis at both time points. Similarly, the expression of OCN was the highest on PCM-2 at both day 14 and day 21, while in addition, both PCM-1 and PCM-3 showed higher values than PCL and the tissue culture control. At day 14, the only significant difference was observed between PCM-2 and PCM-1, while at day 21, PCM-2 again showed the highest OPN expression among all materials, and PCL and tissue culture control were higher than PCM-1 and PCM-3. Finally, the relative protein levels of COL-I in PCM-2 group were the highest at both time points and that in PCM-3 showed significantly higher values than the control and PCL. Collectively, these results further confirmed that the PCM membranes, and in particular PCM-2 supported the osteogenic differentiation of hBMSCs to a significantly larger extent than the PCL membrane without MC.

### Cranial defect healing *in vivo*

Although the evaluation of biomaterials by using hBMSCs could provide clinically relevant results, *in vivo* studies still remain important in the development of implantable medical devices. A critical-size cranial defect model in rats was used to evaluate the bone regenerative performance of the developed guided bone regeneration membranes *in vivo*. PCM-1, PCM-2 and PCM-3 were used as treatment groups, and PCL and an empty defect (blank) served as controls. The samples were harvested at 4 and 12 weeks and analyzed by the means of micro-computed tomography (μ-CT) and histology. μ-CT 3D reconstructed images taken after 4 weeks of implantation showed that in all groups the edges of the defect could still be distinguished, however, no space was observed between the host bone bed and the implants, suggesting a good fit and precise placement of the membranes inside the defects. At 12 weeks after implantation, the boundaries between the materials and the surrounding cranial bone were not as apparent as after 4 weeks, indicating the formation of new bone tissue at the implant periphery and ingrowth into the membrane. In contrast to defects filled with PCM membranes, limited new bone formation occurred in the empty defect and the defect filled with the PCL membrane.

Additionally, the quantitative μ-CT data are shown in Fig.  8. The volume of newly formed bone (BV/TV) and BMD in defects filled with PCM-3 membrane were significantly higher than in the empty defect and the PCL group at both 4 and 12 weeks. Moreover, PCM-3 showed a significantly higher BV/TV and BMD values than PCM-1 at 4 weeks and higher than PCM-1 and PCM-2 at 12 weeks.

**Figure 7.**
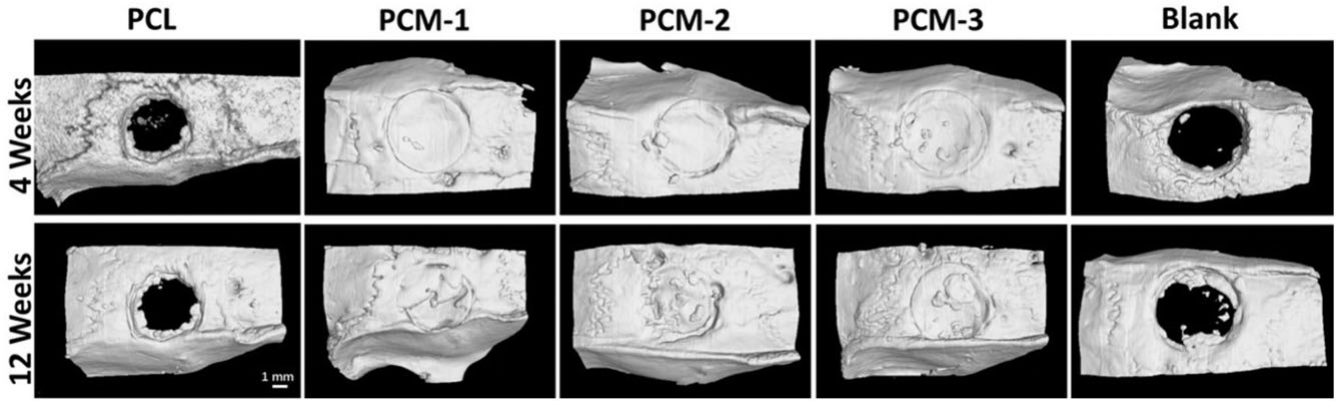
The *in vivo* osteogenesis properties of the membranes. Micro-CT images of cranial defect area treated with PCL, PCM-1, PCM-2, PCM-3 membranes and blank (with defect but without implantations) at 4 and 12 weeks.

**Figure 8.**
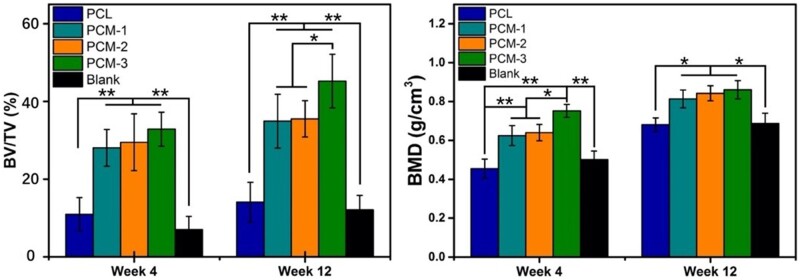
Quantitative analysis of micro-CT data. The bone mass (BV) indicates the specific new bone volume of the defect area and bone mineral density (BMD) exhibiting the bone density of the defect region, (*P < 0.05, **P < 0.01).

**Figure 9.**
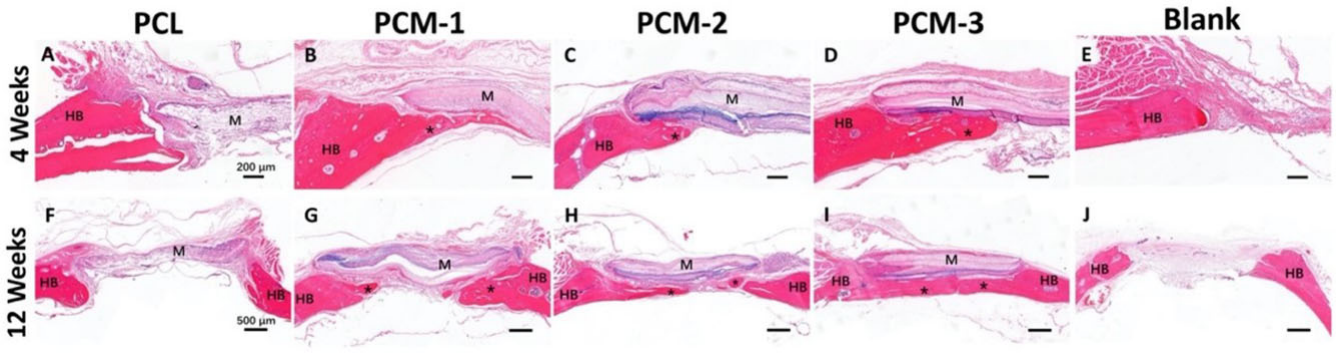
H&E stained histological sections of the defect area treated with different membranes or left empty after 4 and 12 weeks. HB represents host bone, M represents the implanted membranes, asteroids represent new bone. Scale bar = 200 µm (**A–E**), scale bar = 500 µm (**F–J**).

**Figure 10.**
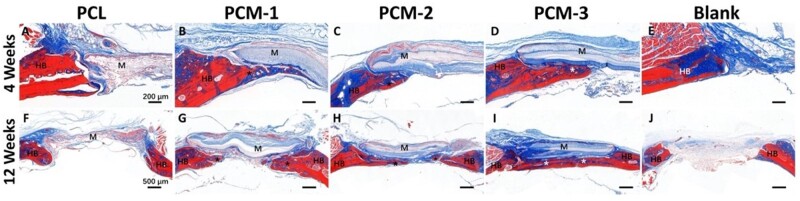
Masson’s trichrome stained histological sections of the defect area treated with different membranes or left empty after 4 and 12 weeks. HB represents host bone, M represents the implanted membranes, asteroids represent new bone. Scale bar = 200 µm (**A–E**), scale bar = 500 µm (**F–J**).

Figures  9 and 10 exhibit images of the histological sections after 4 and 12 weeks of implantation, stained with H&E and Masson’s trichrome, respectively. In the PCL group (Fig.  9A and F), extensive fibrous tissue formation was observed in the defect area, with limited integration between the material and bone defect edge. Comparatively significantly more tissue formation, including bone, was observed in defects with implanted PCM membranes (Fig.  9B–D and G–I). At 4 weeks, a stable connection between the host bone bed and the implanted PCM membranes was observed in the peripheral areas of the defect. Limited new bone formation was also observed underneath the membranes without obvious differences between the three membranes (Fig.  9B–D). At week 12, significantly more bone formation was observed underneath the implanted PCM membranes, the extent of which was the highest in the case of PCM-3, leading to a complete defect bridging, which is in accordance with μ-CT data (Figs.  7 and 8). In the case of defects that did not receive an implant, only infiltration of connective tissue was observed over a period of 12 weeks (Fig.  9E and J).

Figure  10 shows histological sections stained with Masson’s trichrome staining, with collagen stained in blue and mature bone in red. In the PCL group, collagen was observed in the area between the membrane and the surrounding bone. In contrast, little collagen was observed deeper in the defect area, which was predominantly filled with connective tissue (Fig.  10A and F). In the case of defects filled with PCM membranes, the amount of collagen and bone was significantly higher than in the case of PCL, in particular in the PCM-3 group, which is in accordance with the results of the H&E staining. In the empty defect group, a small amount of collagen formed at the margins of the defect accompanied by formation of large amount of fibrous tissue in the defect area (Fig  10E and J).

**Figure 11.**
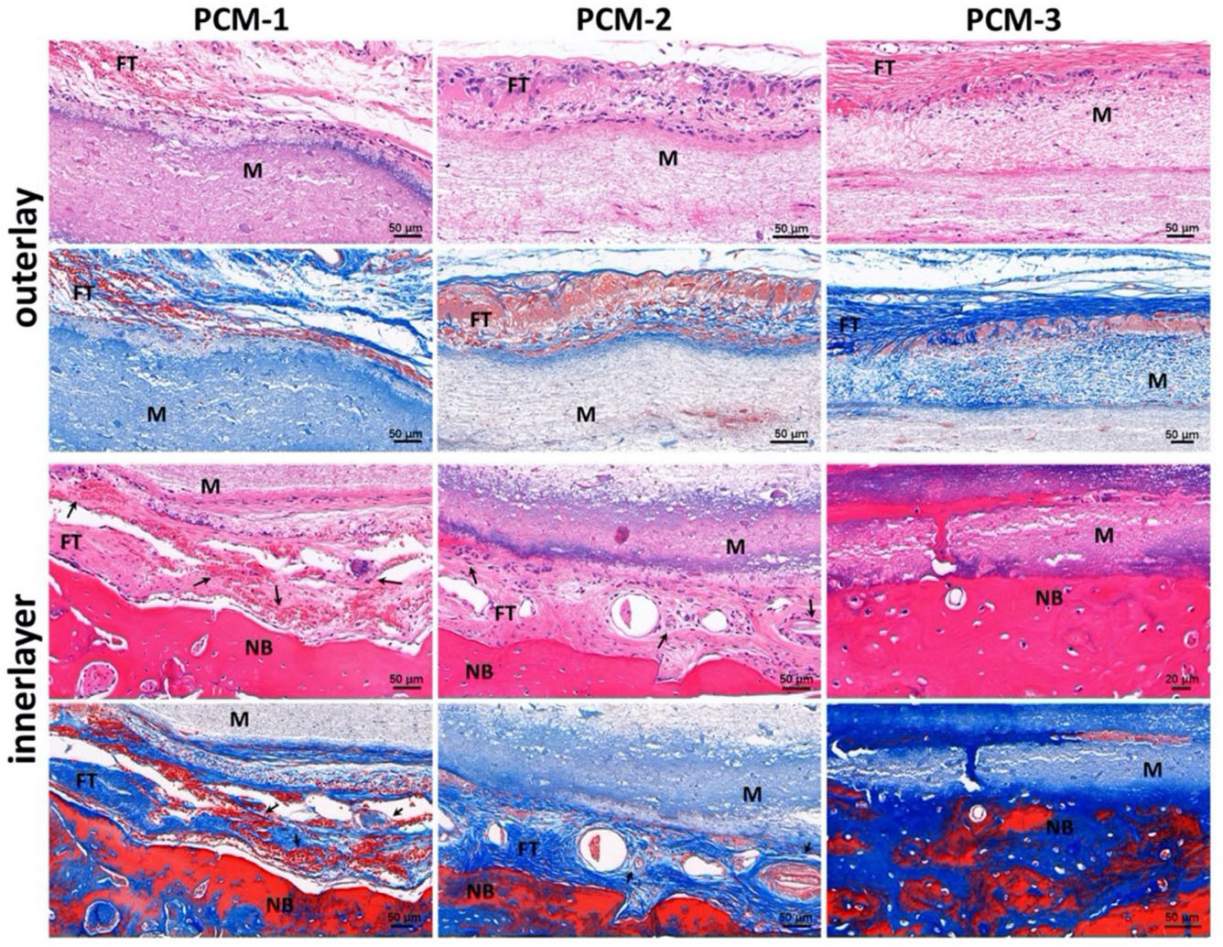
Magnified H&E and Masson stained histological sections of the interface areas in different implanted PCM membranes after 12 weeks. FT represents fibrous tissue, M represents the implanted membranes, NB represents new bone, arrows indicate the blood vessels.

The cell infiltration and cell infiltration shielding capacity of the three-layered membrane are important for it to act as an artificial periosteum, preventing ingrowth of surrounding fibrous tissue on the outer side, while supporting ingrowth and osteogenic differentiation of MSCs on the inner side. Generally, while PCL is considered biocompatible, its hydrophobic nature and lack of cell-recognition sites limit cell adhesion, migration and proliferation in tissue engineering applications. However, these properties are useful when PCL nanofiber mats are applied as a barrier against fibrous tissue infiltration. Figure  11 exhibits magnified edge areas of the implant, showing that few cells reached the middle layer through the outer PCL layer in all PCM membranes. This indeed demonstrates the capacity of the membrane to prevent infiltration of fibrous tissue into the defect area. In the case of PCM-1, abundant blood vessels and collagen fibers were observed at the interface between the implant and host tissue, which is considered to serve as a template for deposition of bone matrix [36, 37]. Nevertheless, no direct tight bond was observed between the membrane and the bone tissue and the amount of new bone that formed inside the membrane was relatively limited. Moreover, the newly formed bone appeared immature. This was in contrast with PCM-2 membranes, where, as shown by the Masson’s trichrome staining, the deposited collagen was more extensive and better organized, and the formed bone tissue appeared more mature. The PCM-3 exhibited the most pronounced osteointegration and osteoconduction, with tight bond between the membrane and the bone tissue and with newly formed mature bone occupying the inner layer of the membrane. These results suggested that the multilayered design of PCM membranes not only provided an environment for new bone formation on the inner side of the membrane but also acted as a barrier for cell and fibrous tissue infiltration on the outer side of the membrane. Besides, the MC content determined the bone regenerative ability of the membranes. Finally, the results showed that the PCM membranes had space-maintaining ability.

Taken together, the histological and μ-CT analyses suggested that PCM-3 membrane was the most efficient in regenerating the critical-size cranial defect in rats. This result is consistent with the *in vitro* observation that membranes containing MC were more favorable for supporting osteogenic differentiation of hBMSCs and revealed the key role for the multilayer structure of the membrane. Nevertheless, while *in vitro*, PCM-2 showed the best performance in terms of support of osteogenic differentiation of hBMSCs, *in vivo*, PCM-3 was the most efficient in healing the cranial bone defects. The exact reason for this inconsistency is difficult to point out because of the many differences between the two experimental setups, but it is plausibly related to the effect the (amount of) mineral has on the surrounding cells *in vitro* and *in vivo*. MC in our membrane was produced using a biomimetic precipitation process, resulting in a poorly crystalline HA, with pronounced ion-exchange dynamics with the environment. Changes in local concentrations of calcium and inorganic phosphate ions have been shown to affect proliferation and osteogenic differentiation of mesenchymal stem cells. For example, Tsai *et al.* [38] found that dissolution of HA particles, resulting in a microenvironment with high calcium ions concentrations caused osteoblasts to immediately switch from the proliferation stage to differentiation stage. They also reported that dissolution of strontium-substituted HA nanofibers resulting in high concentration of calcium and strontium ions in the cell culture medium, positively affected the proliferation and osteogenesis of osteoblasts [22]. Li *et al.* [39] showed that moderate, rather than high concentration of HA nanorods promoted proliferation and expression of osteogenic genes of rat MSCs. Collectively, these data suggest that the positive effect of calcium (and inorganic phosphate) ions on osteogenic differentiation of MSCs is concentration dependent. In our study, it is plausible that PCM-2 with moderate content of MC was favorable for cell proliferation and osteogenic differentiation. *In vivo*, materials were implanted in a more complex microenvironment with stronger buffering effect than in cell culture conditions, possibly explaining why PCM-3 rather than PCM-2 was the best performing material.

In sum, the electrospinning method is an efficient and low-cost method to fabricate artificial periosteum with predesigned composition and structure. By mimicking the natural periosteum multilayered structure and introducing MC in the membrane, cell migration and osteogenic differentiation as well as bone formation were facilitated. Furthermore, the multilayered structure also played a role in shielding the soft tissue from growing into the bone defect area. It is worth mentioning that other studies using electrospun materials and/or cell-hydrogel constructs also provided favorable environment for bone regeneration, like growth factors-loaded fibrous membranes [40], GelMA [14], cell sheets [41, 42] and cell–PEG construct [43], from which design principles can be used to further improve the performance of biomaterials developed in the current study.

## Conclusion

In this study, we constructed three-layered fibrous membranes comprising PCL, PCL/Col and PCL/Col/MC from the outer to the inner layer for guided bone regeneration. Collective results suggested that the fibrous membranes could support hBMSCs attachment, ingrowth/migration and osteogenic differentiation *in vitro* without soluble osteoinductive factors. In addition, the three-layered membranes showed the ability to regenerate critical-size cranial defects in rats. Owing to the presence of MC and the three-layered structure, the PCM membranes promoted the formation of new bone and prevented the infiltration of the surrounding fibrous tissue. Based on these results, the fibrous membrane can be a good candidate for cell delivery and guided bone regeneration.

## Funding

This work was financially supported by National Key R&D Program of China (2017YFC1105000), National Natural Science Foundation of China (51572087), Outstanding Scholar Program of Guangzhou Regenerative Medicine and Health Guangdong Laboratory (2018GZR110102001), GDST-NWO Science Industry Cooperation Program Chemistry (2018A50501006), the 111 Project (B13039). P.H. and Y.Z. acknowledge the financial support by the Gravitation Program ‘Materials Driven Regeneration’, funded by the Netherlands Organization for Scientific Research (NWO) (Grant #024.003.013). J.L. and P.H. acknowledge financial support by the NWO, Applied and Engineering Sciences (NWO-AES, Grant #16711).

*Conflict of interest statement.* The authors declare no conflict of intrest.

**Table 1.** An overview of membranes and their composition

Samples	Sample description	Samples abbreviation	Inner layer content (w/v%)		
			PCL	MC	Col
1	Single layer (layer A)	PCL	10%	0	0
2	Single layer (layer B)	PCL-C	7.5%	0	2.5%
3	Single layer (layer C)	PCM-0	4%	4%	4%
4	Three-layered (A/B/C’)	PCM-1	5%	2.5%	2.5%
5	Three-layered (A/B/C″)	PCM-2	3.3%	3.3%	3.3%
6	Three-layered (A/B/C)	PCM-3	4%	4%	4%
